# IgA Nephropathy with Macroproteinuria and a GFR of 20-30 ml/min/1.73 m^2^ May Still Benefit from RAS Inhibition

**DOI:** 10.1155/2022/9162427

**Published:** 2022-12-30

**Authors:** Ying Wang, Shimin Jiang, Guming Zou, Li Zhuo, Wenge Li

**Affiliations:** ^1^Department of Nephrology, China-Japan Friendship Hospital, Beijing 100029, China; ^2^Graduate School of Chinese Academy of Medical Sciences and Peking Union Medicine College, Beijing 100730, China

## Abstract

**Introduction:**

There has been controversy about renin-angiotensin system (RAS) inhibition in IgAN patients with advanced (stage 4) chronic kidney disease (CKD). Therefore, we investigated the effect of RAS blockade in these patients.

**Methods:**

Renal specimens of 50 IgAN patients who underwent renal biopsy during stage 4 CKD between 2010 and 2020, were stained using immunohistochemistry to detect the expression of RAS receptors (AT1R, AT2R, MasR, and MrgD). The primary endpoint was a composite of end-stage renal disease (ESRD) and death. Main baseline information and the administration of angiotensin-converting enzyme inhibitor (ACEI) or angiotensin II receptor blocker (ARB) were collected.

**Results:**

During a median follow-up time of 25.5 months, 21 (42.0%) patients reached ESRD and none died. Six patients had a baseline eGFR of 15-20 ml/min/1.73m^2^, and reached ESRD with a median renal survival time of 7.0 (range 6.0-23.0) months. Among patients with a baseline eGFR of 20-30 ml/min/1.73m^2^, the percentage of patients using ACEI/ARB in progressive group was much lower than that in stable group (33.3% vs. 62.1%, *P* = 0.045), together with a shorter renal survival time in progressive group (26.0 vs. 30.5 months, *P* = 0.033). Macroproteinuria (24 h − UP ≥ 2.5 g) was also associated with a shorter renal survival time, as well as a significant decline in eGFR of stable group (24.4 vs. 26.4 ml/min/1.73 m^2^, *P* = 0.026). Lower eGFR [hazards ratio (HR), 0.829, 95% confidence interval (CI), 0.724-0.950; *P* = 0.007] and use of ACEI/ARB (HR, 0.356, 95% CI, 0.133-0.953; *P* = 0.040) were predictive of time to ESRD in this stage. No differences were found in the expression of AT1R, AT2R, MasR, and MrgD of renal tissues at the time of biopsy between stable and progressive groups.

**Conclusion:**

Contingent on monitoring serum creatinine and potassium levels, IgAN with macroproteinuria and a GFR of 20-30 ml/min/1.73m^2^ may still benefits from intrarenal RAS inhibition.

## 1. Introduction

IgA nephropathy (IgAN) is the most common primary glomerulonephritis worldwide and still remains a leading cause of end-stage renal disease (ESRD) [1]. A big challenge for clinicians is its heterogeneous risk of progressive kidney function decline, with up to 50% of affected patients progressing to ESRD in their lifetime [2]. Most commonly, IgAN is asymptomatic at the early stage and when diagnosed, some patients have already suffered different degrees of renal injury. Unlike the majority of glomerular diseases, treatment of IgAN is focused on nonimmunosuppressive-based strategies including lifestyle modification, blood pressure control, and optimal inhibition of renin-angiotensin system (RAS) [3].

It is generally accepted that local overactivation of RAS in kidneys plays a necessary role in pathogenesis of IgAN. Both angiotensin-converting enzyme inhibitor (ACEI) and angiotensin II receptor blocker (ARB), collectively called RAS inhibitors (RASi), are now widely used in nearly all kinds of glomerulonephritis because of the renoprotective effect. Many physicians hesitate to use RAS-blocking mediations in patients with advanced (stage 4) chronic kidney disease (CKD), given the concern about the reducing effects of ACEI/ARB on glomerular perfusion and potassium excretion. Consequently, the renal outcome and the risk-benefit profile of RAS inhibitors in this population remain poorly defined [4].

Our team has previously studied the role of conventional therapy in patients with advanced diabetic nephropathy (DN) [5]. Based on this thought, to investigate if ACEI/ARB could continue to delay the onset of ESRD in the late stage of IgAN, we therefore conducted this study to detect the expression of four RAS receptors and the effects of ACEI/ARB in IgAN patients with stage 4 CKD.

## 2. Methods

### 2.1. Study Design and Participants

**Figure 1:**
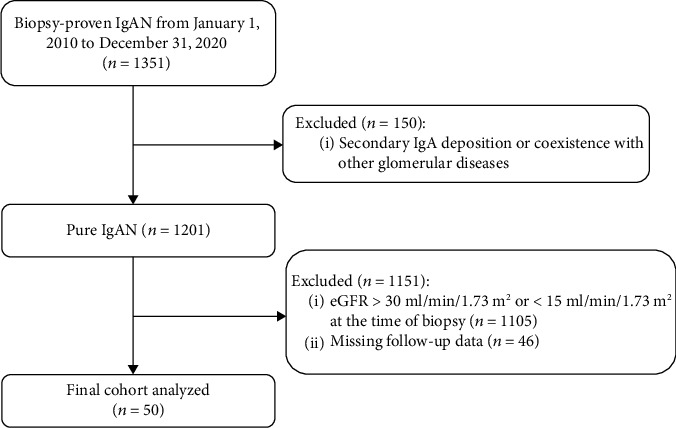
Flowchart of study participants. IgAN: IgA nephropathy, eGFR: estimated glomerular filtration rate, CKD: chronic kidney disease.

This was a retrospective study. We enrolled patients diagnosed of IgAN who underwent a renal biopsy at China-Japan Friendship Hospital from January 1, 2010 to December 31, 2020. The renal biopsy was operated by clinicians with many years of relevant experience in our department. Inclusion criteria were as follows: (1) biopsy-proven IgAN patients with stage 4 CKD at the time of biopsy; (2) age from 18 to 75 years. The main exclusion criteria were: patients (1) with secondary IgA deposits including purpuric nephritis, lupus nephritis, or coexistence with other glomerular diseases; (2) those without follow-up data (Figure 1).

This study was approved by the ethics committee of the China-Japan Friendship Hospital (2021-113-K71). As this was a retrospective observational study of deidentified data, patient informed consent was not required. All the procedures that included human participants adhered to the Declaration of Helsinki.

### 2.2. Parameters and Definitions

The following clinical parameters were collected from the electronic medical system: gender, age, mean arterial pressure (MAP), 24 h urinary protein excretion (24 h-UP), urinary red blood cell count (URBC,/HPF, the most before renal biopsy), serum creatinine (SCr), hemoglobin (Hb), total cholesterol (TC), triglyceride (TG), parathyroid hormone (PTH), and uric acid (UA). We calculated estimated glomerular filtration rate (eGFR) using the creatinine-based Chronic Kidney Disease Epidemiology Collaboration (CKD-EPI) equation [6]. Postbiopsy use of RAS blockade or immunosuppressive (IS) therapies were also retrospectively reviewed. Hypertension was defined as systolic blood pressure (SBP) ≥140 mmHg and (or) diastolic blood pressure (DBP) ≥90 mmHg.

### 2.3. Endpoints and Follow-Up

The primary endpoint was a composite of ESRD and death. ESRD was defined as the initiation of maintenance dialysis or renal transplantation. A three-year follow-up was conducted. The progressive group was defined as those with the occurrence of the endpoint events, and the stable group was patients whose renal function was still maintained in stage 4 CKD.

### 2.4. Renal Biopsy and Immunohistochemistry (IHC) of RAS Receptors

The antibody used in this study was the anti-angiotensin II type 1 receptor (AT1R) antibody (Abcam, ab124505, 1: 500), anti-angiotensin II type 2 receptor (AT2R) antibody (Abcam, ab254561, 1: 400), anti-Mas receptor (MasR) antibody (Abcam, ab66030, 1: 100), and anti-Mas associated G protein coupled receptor D (MrgD) antibody (Invitrogen, PA5-100957, 1: 400). Paraffin-embedded tissues were sectioned at 3 *μ*m thickness. After dewaxing and hydration treatment, heat-mediated antigen retrieval was performed for all the receptors. The sections for staining AT1R and MasR were treated with citrate buffer while those for staining AT2R and MrgD were treated with ethylene diamine tetraacetic acid (EDTA) buffer [Gene Tech (Shanghai) Co., Ltd, GT100411, 1: 50]. After immunostaining, all sections were counterstained with hematoxylin. Immunohistochemical images were obtained using a Moticam 2506 instrument (Motic, Fujian, China) from sections observed microscopically at ×400 magnification (Nikon, Tokyo, Japan). The images were acquired using an integrated digital camera system (Nikon). Immunoreactivity was semiquantitatively evaluated in a blinded manner. Image Pro-plus (IPP) computer image analysis software (Media Cybernetics, Bethesda, MD, USA) was used to analyze the pixel density of the stained areas, and to quantify protein levels.

### 2.5. Statistical Analysis

Continuous data were regarded as nonparametric and were presented as median and interquartile range (IQR). Differences were analyzed using the Mann–Whitney *U* nonparametric test. Categorical variables were reported as percentages and analyzed using the chi-square or Fisher's exact test. Univariate and multivariate Cox proportional hazard model was used to calculate the hazard ratios (HRs) and 95% confidence intervals (CIs) for variables related to the composite outcome. Statistical analysis was performed using SPSS software (version 24.0; IBM Corp, Armonk, NY) and GraphPad Prism 8.0. Two-sided *P* values <0.05 were considered to indicate statistical significance.

## 3. Results

### 3.1. Baseline and Pathological Characteristics

We totally enrolled 50 IgAN patients with stage 4 CKD at the time of biopsy aged 18-75 years, who underwent renal biopsy in the China-Japan Friendship Hospital from 2010 to 2020. The clinical characteristics of the patients are presented in Table 1. The median age of all patients was 40.5 (IQR 29.8-49.3) years, and 24 (48.0%) patients were male. The median eGFR was 25.8 (23.1-26.8) ml/min/1.73m^2^. The median 24 h-UP was 2.7 (1.5-4.3) g. By the end of the follow-up, 21 (42.0%) of the 50 patients progressed to ESRD. None died or developed hyperkalemia (serum potassium ≥ 6.0 mmol/l) or acute kidney injury (AKI) requiring hospitalization [5]. At baseline, there was no significant difference in main biochemical parameters such as Hb (119.0 vs. 107.0 g/l, *P* = 0.075), eGFR (25.7 vs. 24.3 ml/min/1.73m^2^, *P* = 0.072), and 24 h-UP (2.0 vs. 3.0 g/d, *P* = 0.149) between the progressive group and stable group. Similarly, there was also no difference in the main parameters between the two groups if stratified by use of RASi (Supplementary Table 1). Although there was a significant difference in the time of follow-up, 18 (62.1%) patients in the stable group received RAS blockade therapy after biopsy while the percentage in progressive group was 33.3% (*P* = 0.045). This difference could also be found between using RASi group and without RASi group (Supplementary Table 1). There were 15 (of 50) patients using RASi prior to biopsy, but they all use the drugs very irregularly or just for a short time (no more than 3 weeks).

### 3.2. Follow-Up and Renal Outcomes

**Figure 2:**
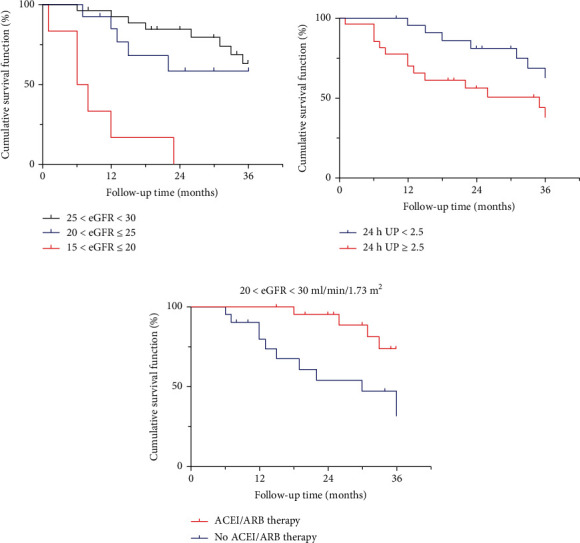
Subgroup analysis suggesting that the mean renal survival time varied according to the baseline eGFR and 24 h-UP. The mean renal survival time was significantly longer in those with a baseline eGFR of 20-30 ml/min/1.73 m^2^ and 24 h − UP < 2.5 g ((a, b), *P* < 0.001 and *P* = 0.042, respectively). In patients with a baseline eGFR of 20-30 ml/min/1.73 m^2^, RAS blockade resulted in a prolonged renal survival time ((c), *P* = 0.006).

Fifteen (71.4%) patients in the progressive group had a baseline eGFR of 20-30 ml/min/1.73m^2^, and the other six (28.6%) patients had baseline eGFR values of 15-20 ml/min/1.73m^2^. All 29 patients in the stable group had a baseline eGFR of 20-30 ml/min/1.73m^2^. By the end of follow-up, a significant difference in renal survival time was observed between the progressive and stable groups [24.0 (range 6.0-36.0) vs. 30.5 months (8.0-36.0), *P* = 0.003], and none of the patients in the stable group progressed to the endpoint. The median renal survival time of patients in the progressive group with a baseline eGFR of 15-20 ml/min/1.73 m^2^ was 7.0 (6.0-23.0) months, much shorter than that in patients with a baseline eGFR of 20-30 ml/min/1.73 m^2^ (*P* < 0.001, Figure 2(a)). Furthermore, the median renal survival time among all patients whose baseline eGFR was 20-30 ml/min/1.73 m^2^ was significantly different between the progressive and stable groups (26.0 vs. 30.5 months, *P* = 0.033). Yet, no decline in renal function was observed in the stable group at the end of follow-up compared to the baseline level (24.4 vs. 25.7 ml/min/1.73 m^2^, *P* = 0.414). These results suggest that a lower baseline eGFR indicated worse renal survival in IgAN patients with stage 4 CKD. We then analyzed ACEI/ARB use in the two groups. One third of the patients in the progressive group used an ACEI/ARB, which was much lower than the percentage in the stable group (62.1%, *P* = 0.045). With a baseline eGFR of 20-30 ml/min/1.73 m^2^, those not receiving ACEI/ARB therapy shared a median renal survival time of 30.0 months (*P* = 0.006). The results were shown in Figure 2.

We separated the patients into two subgroups according to the baseline 24 h-UP level, with a threshold value of 2.5 g/d. The same was applied to the stable group. The mean survival times of patients with 24 h − UP < 2.5 g and ≥ 2.5 g in the progressive group were 30.0 and 24.5 months, respectively (*P* = 0.043). A notable decrease in eGFR was detected in the stable group by the end of follow-up (24.4 vs. 26.4 ml/min/1.73 m^2^, *P* = 0.026) in those whose 24 h − UP ≥ 2.5 g, while the median eGFR at the end of follow-up and at baseline were 24.0 and 25.0 ml/min/1.73 m^2^ among those with a 24 h − UP < 2.5 g in the stable group (*P* = 0.381). The result indicates that more urinary protein excretion was associated with a shorter renal survival time. Multivariate Cox analysis suggested that the baseline eGFR level (*P* = 0.007), use of RASi (*P* = 0.040) were independent risk factors for renal endpoints in IgAN patients with stage 4 CKD (Table 2).

### 3.3. Angiotensin Receptors Expression and RAS-Blocking Mediation

**Figure 3:**
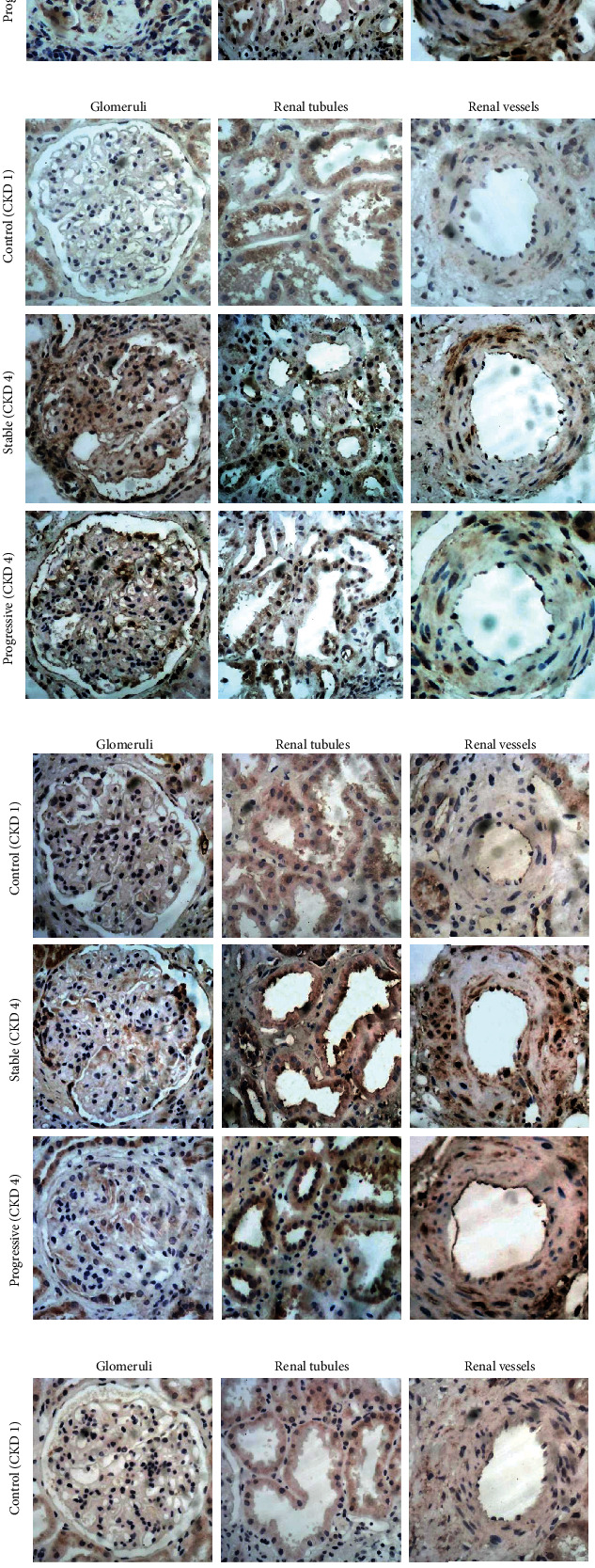
Immunohistochemical staining suggests increased expression of AT1R and AT2R ((a, c) ×400) and decreased expression of MasR ((b), ×400) of IgAN patients with stage 4 CKD in the stable and progressive groups, while MrgD receptor expression was similar ((d), ×400).

**Figure 4:**
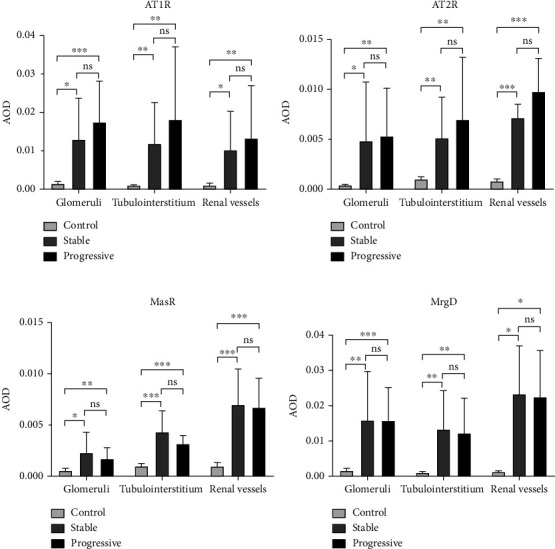
IHC showed a significant increased expression of the four receptors in IgAN patients with stage 4 CKD compared to the control group (IgAN patients with stage 1 CKD who had normal renal function with mild mesangial proliferative glomerulonephritis on biopsy). No significant difference was observed in the expression levels of AT1R (a), AT2R (b), MasR (c), and MrgD receptors (d) in renal biopsy specimens between stable and progressive groups. AOD, average optical density. ∗*P* < 0.05; ∗∗*P* < 0.01; ∗∗∗*P* < 0.001; ns, no significance.

Overactivation of the RAS plays an important role in the pathogenesis of IgAN, and our IHC results support this statement. IHC staining showed that the expression of the angiotensin receptors (AT1R, AT2R, MasR, and MrgD) increased significantly in IgAN renal tissues of stage 4 CKD, whether progressive or stable group, compared to those with stage 1 CKD (mild mesangial proliferative glomerulonephritis) at the time of renal biopsy. Patients in progressive group exhibited a higher expression of AT1R and AT2R and lower expression of MasR, while the expression of MrgD receptor was similar in the two groups. However, all the differences of four receptors between the progressive and stable groups were not significant. The expression of the four receptors is shown in Figures 3 and 4.

## 4. Discussion

IgAN is now still the most common primary glomerulonephritis worldwide. Patients may suffer various degrees of renal insufficiency at the time of diagnosis. With a severe renal injury, patients with stage 4 CKD often have already lost the opportunities of renal biopsy for a definite diagnosis, leading to a limited clinical and pathological data. RAS inhibition is now one of the main therapies for IgAN but remains controversial in those with severe renal injury. To our knowledge, this is the specific study demonstrating the effects and prognostic relevance of ACEI/ARB in advanced (stage 4 CKD) IgAN patients. Since current information indicates that most of patients with stage 4 CKD are not offered such renal protective treatment, our results may have important implications for the development of new therapeutic guidelines.

The role of renal RAS overactivation has been long highlighted in the onset of IgAN. Our previous studies also supported the statement [7]. AT1, AT2, Mas, and MrgD receptors are the main four receptors for angiotensin. AT1R usually mediates vasoconstriction, cell proliferation, and renal fibrosis, which is the main target of ARB drugs. The molecular and cellular actions of angiotensin II in cardiovascular and renal diseases are almost exclusively mediated via the AT1R [8]. AT1R inhibition reduces blood pressure and has anti-inflammatory and antiproliferative effects, while an increasing number of publications emphasize the role of AT2R in the so-called protective arm of RAS [9]. Besides, MasR is also regarded as a kind of protective receptor as angiotensin (1-7), which functions as a vasodilator and antiproliferative agent, is the physiologic ligand of the MasR. MrgD is a member of Mas-related G protein coupled receptor family. There are only a few studies about the MrgD receptor and its endogenous ligand alamandine, but the structural resemblance of the ligands angiotensin (1-7) and alamandine as well as of the receptors Mas and MrgD suggests a possible role of MrgD in blood pressure regulation and hypertension [10]. So far, there are very few publications demonstrating the correlation between MrgD and IgAN. Our immunohistochemical results indicated that AT1R and AT2R expression were significantly increased in advanced IgAN, whereas a decreased expression of MasR and similar expression of MrgD compared to those with stage 1 CKD at the time of biopsy. However, no differences of the expression of the four receptors were found between progressive and stable groups. This may provide some evidence about MrgD in IgAN.

With a median follow-up time of our patients which was 25.5 months, multivariate Cox regression analysis suggested the baseline eGFR was a strong prognostic factor for IgAN patients with stage 4 CKD. Patients with baseline eGFR <20 ml/min/1.73 m^2^ might rapidly progress to renal end points according to our results. Although there was a difference of renal survival between patients with 24 h − UP ≥ 2.5 g/d and < 2.5 g/d, 24 h-UP is not observed as a risk factor for IgAN patients with stage 4 CKD according to our study. This may be due to the relatively limited sample size and the more severe renal injuries of patients in our study at the time of biopsy. In recent years, the prognostic significance of hematuria in IgAN has drawn people's attention [11, 12], while there was no significant correlation between hematuria and prognosis in this study, probably because our focus was on patients with badly impaired renal function.

For IgAN patients with macroproteinuria and a GFR of <30 ml/min/1.73 m^2^, despite glucocorticoids and some novel immunosuppressants [13, 14], the 2021 KDIGO guideline no longer recommends immunosuppression in these patients as it may do more harm than good [3]. So far, RAS blockade is still the first-line treatment of IgAN and even almost other kinds of glomerulonephritis. Increased risk of hyperkalemia or elevating serum creatinine discourages the use of RAS-blocking mediation in those with advanced CKD. However, there are some stage 4 CKD patients benefiting from ACEI/ARB in trials, including the AIPRI [15], ESBARI [4], STOP-IgAN [16], and TESTING [17] trials. Angiotensin receptors, especially AT1R, were still upregulated in these patients, supporting the use of RAS inhibition. Consequently, a total of 25 patients received RAS blockade after biopsy, 7 (33.3%) in progressive group and 18 (62.1%) in stable group, respectively. All of these patients shared the baseline eGFR of 20-30 ml/min/1.73m^2^. Our results suggested the use of an ACEI/ARB was predictive of the time to ESRD for IgAN patients with macroproteinuria and a GFR of 20-30 ml/min/1.73m^2^. Therefore, AECI/ARB may still bring a renal benefit for advanced (stage 4 CKD) IgAN in a 3-year follow-up.

Nevertheless, there are several limitations of our study. First, due to the severe renal injury and the high risk of postbiopsy complications, such as bleeding, patients with stage 4 CKD were seldom biopsied for a definite diagnosis. Therefore, our study may mean something for the precious data of these patients. Yet, our findings still need to be confirmed in a multicenter study with a larger sample size. Moreover, this is a retrospective study and presents methodologic limitations that must be taken into account, so causality was difficult to infer. Proportional hazards regression incorporating time-varying covariates may create time-varying confounding. Besides, we included cases during ten years, so it was a difficulty for us to find the data of all the patients prior to biopsy.

## 5. Conclusion

In summary, ACEI/ARB therapy may still be considered in IgAN patients with macroproteinuria and a GFR of 20-30 ml/min/1.73m^2^, contingent on monitoring serum creatinine and potassium levels.

## Figures and Tables

**Table 1:** Clinical baseline of IgAN patients with stage 4 CKD.

Clinical parameters	Total (*n* = 50)	Stable (*n* = 29)	Progressive (*n* = 21)	*P* value
*Clinical findings*				
Age (y)	40.5 (29.8-49.3)	43.0 (29.5-58.0)	37.0 (29.5-44.5)	0.132
Gender, M (%)	24 (48.0)	16 (55.2)	8 (38.1)	0.233
SBP (mmHg)	135.0 (122.0-147.0)	130.0 (121.0-139.5)	141.0 (125.5-151.0)	0.205
DBP (mmHg)	85.0 (77.8-96.5)	80.0 (76.0-90.0)	93.0 (79.0-100.0)	0.061
MAP (mmHg)	100.2 (95.2-113.0)	98.7 (90.5-103.0)	107.33 (97.2-116.0)	0.080
Hypertension, *n* (%)	30 (60.0)	17 (58.6)	13 (61.9)	0.815
Use of RASi, *n* (%)	25 (50.0)	18 (61.2)	7 (33.3)	**0.045**
Use of IS, *n* (%)	25 (50.0)	17 (58.6)	8 (38.1)	0.152
*Laboratory findings*				
Hb (g/l)	115.5 (103.8-125.3)	119.0 (110.0-126.5)	107.0 (97.0-120.0)	0.075
eGFR (ml/min/1.73m^2^)	25.8 (23.1-26.8)	25.7 (24.1-27.6)	24.3 (19.7-26.6)	0.072
UA (*μ*mol/l)	478.5 (440.5-536.8)	477.0 (450.0-525.5)	480.0 (410.0-574.5)	0.891
iPTH (pg/ml)	76.8 (50.5-136.8)	68.0 (47.5-99.5)	91.4 (58.2-192.3)	0.071
TC (mmol/l)	5.0 (4.1-6.2)	4.8 (4.0-6.1)	6.0 (4.2-6.7)	0.135
TG (mmol/l)	1.8 (1.6-2.4)	1.8 (1.5-2.9)	2.0 (1.6-2.3)	0.340
24 h-UP (g)	2.7 (1.5-4.4)	2.0 (1.5-4.5)	3.0 (1.8-4.4)	0.149
URBC (/HPF)	11.2 (4.7-36.9)	11.5 (4.3-31.1)	10.2 (4.6-42.4)	0.992
Follow-up time (months)	25.5 (12.0-36.0)	36.0 (22.0-36.0)	15.0 (7.5-28.5)	<0.001

IgAN, IgA nephropathy; SBP, systolic blood pressure; DBP, diastolic blood pressure; MAP, mean arterial pressure; RASi, renin-angiotensin system inhibitor; IS, immunosuppressive agents; Hb, hemoglobin; eGFR, estimated glomerular filtration rate; UA, uric acid; PTH, parathyroid hormone; TC, total cholesterol; TG, triglyceride; 24 h-UP, 24 h urinary protein excretion; URBC, urinary red blood cells.

**Table 2:** Univariate and multivariate Cox regression analysis of IgAN patients with stage 4 CKD.

Variables	Univariate analysis		Multivariate analysis	
	HR (95% CI)	*P*	HR (95% CI)	*P*
Age (y)	0.962 (0.925-1.001)	0.055		
Gender	1.496 (0.619-3.615)	0.371		
SBP	0.998 (0.969-1.029)	0.912		
DBP	1.023 (0.976-1.073)	0.343		
MAP	1.018 (0.989-1.046)	0.223		
Hb	0.977 (0.949-1.006)	0.122		
eGFR	0.801 (0.696-0.921)	0.002	0.829 (0.724-0.950)	**0.007**
UA	0.998 (0.992-1.004)	0.554		
iPTH	1.001 (0.999-1.002)	0.526		
TC	1.178 (0.902-1.539)	0.230		
TG	1.089 (0.771-1.537)	0.629		
URBC	0.999 (0.994-1.003)	0.569		
24 h-UP	1.157 (0.962-1.391)	0.121	1.108 (0.922-1.331)	0.275
Hypertension	1.017 (0.421-2.460)	0.970		
RASi	0.259 (0.103-0.653)	0.004	0.356 (0.133-0.953)	**0.040**
IS	0.608 (0.252-1.466)	0.268		

SBP, systolic blood pressure; DBP, diastolic blood pressure; MAP, mean arterial pressure; RASi, renin-angiotensin system inhibitor; IS, immunosuppressive agents; Hb, hemoglobin; eGFR, estimated glomerular filtration rate; UA, uric acid; PTH, parathyroid hormone; TC, total cholesterol; TG, triglyceride; 24 h-UP, 24 h urinary protein excretion; URBC, urinary red blood cells.

## Data Availability

All data are available and displayed in our article.

## Supplementary Materials

Supplementary Materials Supplementary Table 1: showed the clinical baseline of IgAN patients with stage 4 CKD stratified by use of RASi. No significant difference was found in the main parameters between the two groups.
