# Supplementary material for: IgA Nephropathy with Macroproteinuria and a GFR of 20-30 ml/min/1.73 m2 May Still Benefit from RAS Inhibition
Source: J Renin Angiotensin Aldosterone Syst. 2022 Dec 30;2022:9162427. doi: 10.1155/2022/9162427 (PMC9822756; doi:10.1155/2022/9162427)
Supplement: Supplementary Materials — Supplementary Table 1: showed the clinical baseline of IgAN patients with stage 4 CKD stratified by use of RASi. No significant difference was found in the main parameters between the two groups. [file 9162427.f1.docx]

**Supplementary Table 1** Clinical Baseline of IgAN patients with stage 4 CKD stratified by use of RASi

| Clinical Parameters | Total  (n=50) | Without RASi  (n=25) | RASi mediation  (n=25) | *P* value |
| --- | --- | --- | --- | --- |
| Clinical findings | | | | |
| Age (y) | 40.5 (29.8-49.3) | 36.0 (27.0-47.0) | 43.0 (31.5-55.5) | 0.095 |
| Gender, M (%) | 24 (48.0) | 14 (56.0) | 10 (40.0) | 0.258 |
| SBP (mmHg) | 135.0 (122.0-147.0) | 136.0 (126.5-146.0) | 132.0 (117.5-155.0) | 0.705 |
| DBP (mmHg) | 85.0 (77.8-96.5) | 90.0 (80.0-98.0) | 84.0 (73.5-94.0) | 0.303 |
| MAP (mmHg) | 100.2 (95.2-113.0) | 101.6 (97.2-113.0) | 98.3 (88.0-114.5) | 0.347 |
| Hypertension, n (%) | 30 (60.0) | 13 (52.0) | 17 (68.0) | 0.248 |
| Use of IS, n (%) | 25 (50.0) | 15 (60.0) | 10 (40.0) | 0.157 |
| Endpoint, n (%) | 21 (42.0) | 14 (56.0) | 7 (28.0) | **0.045** |
| Laboratory findings | | | | |
| Hb (g/L) | 115.5 (103.8-125.3) | 112.0 (100.0-121.5) | 119.0 (107.0-128.0) | 0.362 |
| eGFR (ml/min/1.73m^2^) | 25.5 (23.1-26.8) | 24.1 (20.7-26.4) | 26.0 (25.0-27.4) | 0.055 |
| UA (μmol/L) | 478.5 (440.5-536.8) | 477.0 (423.5-525.5) | 481.0 (444.0-544.5) | 0.600 |
| TC (mmol/L) | 5.0 (4.1-6.2) | 5.1 (3.7-6.6) | 4.9 (4.2-6.1) | 0.907 |
| TG (mmol/L) | 1.8 (1.6-2.4) | 2.0 (1.6-2.9) | 1.8 (1.5-2.1) | 0.218 |
| 24h-UP (g) | 2.7 (1.5-4.4) | 3.0 (1.7-5.7) | 2.1 (1.5-3.2) | 0.126 |
| URBC (/HPF) | 11.2 (4.7-36.9) | 11.6 (3.9-67.2) | 10.9 (5.0-23.8) | 0.655 |

IgAN, IgA nephropathy; SBP, systolic blood pressure; DBP, diastolic blood pressure; MAP, mean arterial pressure; RASi, renin-angiotensin system inhibitor; IS, immunosuppressive agents; Hb, hemoglobin; eGFR, estimated glomerular filtration rate; UA, uric acid; TC, total cholesterol; TG, triglyceride; 24h-UP, 24h urinary protein excretion; URBC, urinary red blood cells.
